# Cyclic AMP Control Measured in Two Compartments in HEK293 Cells: Phosphodiesterase K_M_ Is More Important than Phosphodiesterase Localization

**DOI:** 10.1371/journal.pone.0024392

**Published:** 2011-09-08

**Authors:** Karina Matthiesen, Jacob Nielsen

**Affiliations:** Synaptic Transmission 2, H. Lundbeck A/S, Copenhagen, Denmark; University of Oldenburg, Germany

## Abstract

The intracellular second messenger cyclic AMP (cAMP) is degraded by phosphodiesterases (PDE). The knowledge of individual families and subtypes of PDEs is considerable, but how the different PDEs collaborate in the cell to control a cAMP signal is still not fully understood. In order to investigate compartmentalized cAMP signaling, we have generated a membrane-targeted variant of the cAMP Bioluminiscence Resonance Energy Transfer (BRET) sensor CAMYEL and have compared intracellular cAMP measurements with it to measurements with the cytosolic BRET sensor CAMYEL in HEK293 cells. With these sensors we observed a slightly higher cAMP response to adenylyl cyclase activation at the plasma membrane compared to the cytosol, which is in accordance with earlier results from Fluorescence Resonance Energy Transfer (FRET) sensors. We have analyzed PDE activity in fractionated lysates from HEK293 cells using selective PDE inhibitors and have identified PDE3 and PDE10A as the major membrane-bound PDEs and PDE4 as the major cytosolic PDE. Inhibition of membrane-bound or cytosolic PDEs can potentiate the cAMP response to adenylyl cyclase activation, but we see no significant difference between the potentiation of the cAMP response at the plasma membrane and in cytosol when membrane-bound and cytosolic PDEs are inhibited. When different levels of stimulation were tested, we found that PDEs 3 and 10 are mainly responsible for cAMP degradation at low intracellular cAMP concentrations, whereas PDE4 is more important for control of cAMP at higher concentrations.

## Introduction

The second messenger adenosine 3′,5′-cyclic monophosphate (cAMP) is involved in a variety of intracellular processes [1]. Most importantly, cAMP regulates the activity of protein kinase A, which in turn activates several downstream targets [2]. cAMP signals are initiated by transmembrane adenylyl cyclases that generate cAMP from ATP when activated by ligand binding to G_s_-coupled GPCRs [3]. cAMP is degraded by phosphodiesterases (PDE) [4]. The 11 families of PDEs comprise numerous subtypes and splice variants, differing in expression pattern, subcellular localization, substrate affinities, and mode of regulation [5]–[7]. Thus, how external stimuli are processed by cells through cAMP depends not only on receptor profile but also on the subtypes of PDEs expressed in any given cell type.

The recent development of genetically encoded FRET sensors for cAMP detection [8] has made direct study of cAMP regulation in living cells possible. In cardiac myocytes, β-adrenergic stimulation generates multiple microdomains of increased cAMP concentration [9]. In primary cultures of hippocampal neurons, FRET sensors have been used to study the propagation of cAMP signals along neurites [10], [11]. FRET sensors have also been used to study cAMP compartmentalization in the much smaller HEK293 cells by targeting FRET sensors to specific subcellular compartments such as plasma membrane, nucleus, or mitochondria [12], [13]. Both studies observed a faster cAMP response at the plasma membrane compared to the cytosol after adenylyl cyclase stimulation. In one of these studies [13], but not in the other [12], the maximal cAMP response at the plasma membrane was also significantly higher than in the cytosol. These and other data suggest that cAMP is in many cases compartmentalized, i.e. that cAMP concentration differs between cellular subdomains under certain conditions. Together with differential subcellular localization of downstream signaling mediators such as protein kinase A isoforms, this is thought to underlie compartmentalization of cAMP signaling [14], [15]. The mechanism for achieving cAMP compartmentalization that has most experimental support is that the rates of cAMP degradation differ between compartments due to subcellularly localized PDEs [14]–[16].

Intracellular measurement of cAMP with FRET sensors requires imaging of single cells. This is time-consuming and limits the number of conditions that can be tested. A recently developed cAMP BRET sensor makes it possible to study populations of cells [17], [18]. While the FRET sensors require excitation of the donor molecule through an external source, the BRET sensor produces the energy required for the donor emission with the encoded luciferase. This leads to a higher signal-to-noise ratio because no autofluorescence is produced.

In the present study, we have generated a membrane-targeted variant of the cAMP BRET sensor CAMYEL [17] and have compared cAMP measurements from it to that of the cytosolic CAMYEL in HEK293 cells. We have analyzed PDE activity in subcellularly fractionated lysates from HEK293 cells. We found that PDE4 dominates cAMP degradation in the cytosol, while PDEs 3 and 10 dominate in the membrane fraction. We have tested a range of concentrations of the direct adenylyl cyclase activator forskolin and the GPCR ligand prostaglandin E1 (PGE1) in combination with selective inhibitors to membrane-bound and cytosolic PDEs. We found no evidence that the membrane-associated and cytosolic PDEs have differential effects on membrane-proximal and cytosolic concentrations of cAMP. When different levels of stimulation were tested, we found that PDEs 3 and 10 are mainly responsible for cAMP degradation at low cAMP concentrations whereas PDE4 is more important for controlling cAMP at higher concentrations.

## Results

### Cytosolic and membrane-targeted cAMP BRET sensors

**Figure 1:**
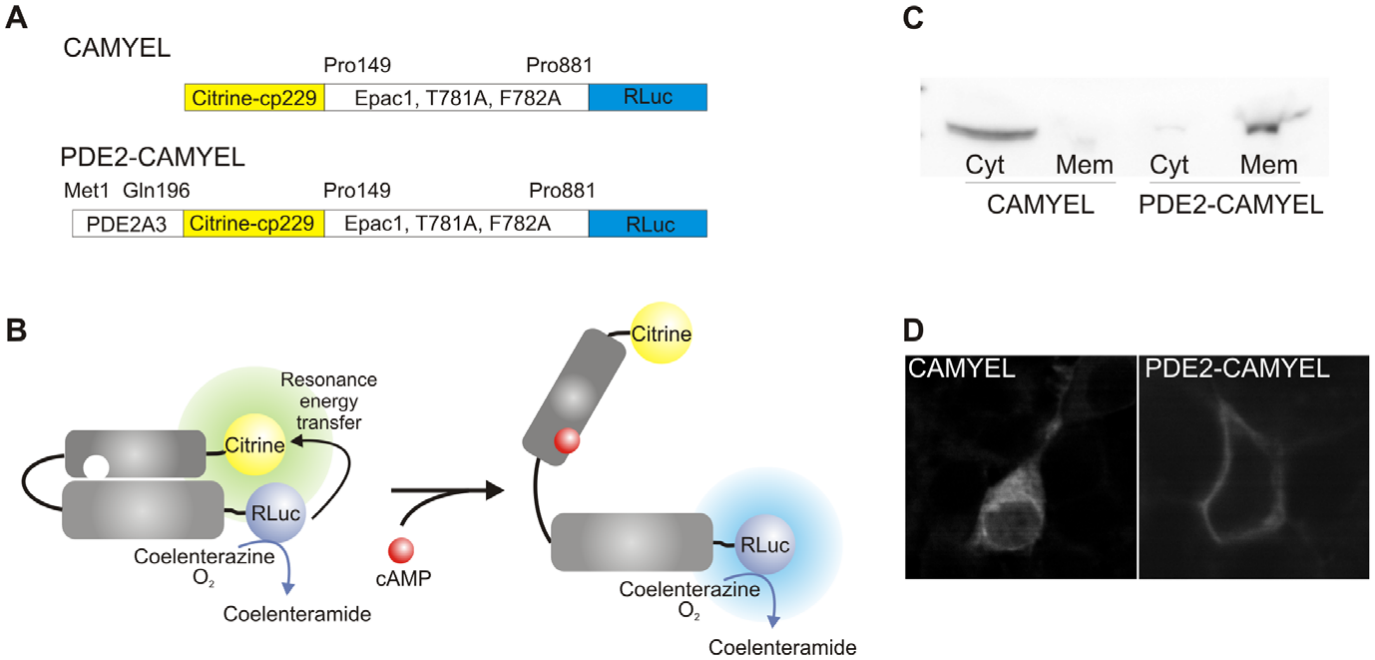
Schematic overview of BRET sensors. (A) CAMYEL is comprised of cytosolic, catalytic inactive Epac1 sandwiched between Citrine-cp229 and the *Renilla* luciferase (RLuc). PDE2-CAMYEL is targeted to the membrane by fusion of the N-terminal part of PDE2A3 to CAMYEL. (B) Binding of cAMP to CAMYEL/PDE2-CAMYEL induces a conformational change in the Epac1 part resulting in a decrease of energy transfer from the luciferase to Citrine. (C and D) Western blot and confocal images showing the distribution of CAMYEL in the cytosol and the targeting of PDE2-CAMYEL to the membrane. Cyt, cytosolic fraction; Mem, membrane fraction.

In order to measure changes of cAMP concentration in real time in living cells we used the cAMP BRET sensors CAMYEL [17] and PDE2-CAMYEL. CAMYEL consists of catalytically inactive Epac1 sandwiched between the *Renilla* luciferase and the yellow fluorescent protein variant Citrine (Figure 1A). The mechanism underlying detection of cAMP by this sensor is schematized in Figure 1B. We found that the CAMYEL sensor is evenly distributed in the cytosol (Figure 1C and D) in agreement with previous observations [17].

In order to also measure cAMP near the plasma membrane, a membrane localized variant of the sensor was needed. The PDE2A splice variant 3 has been shown to be targeted to the plasma membrane through myristoylation and palmitoylation of the N-terminal region [19]. We fused the 196 N-terminal amino acids of this splice variant to the N-terminal of the BRET sensor CAMYEL and so generated the membrane localized variant PDE2-CAMYEL. As shown in Figure 1C and D, this cAMP BRET sensor is targeted to the plasma membrane as predicted.

**Figure 2:**
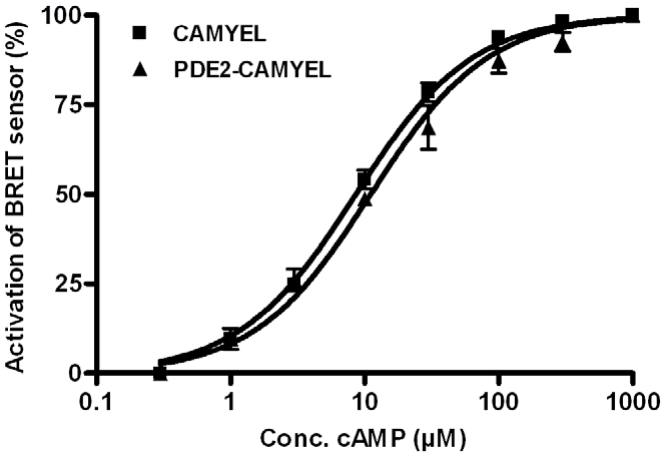
Characterization of the BRET sensors. cAMP dose-response graphs showing the relative activation of CAMYEL and PDE2-CAMYEL from HEK293 lysates. EC_50_ values were 8.4±0.7 µM and 10.6±0.9 µM, respectively (n = 3–4).

For direct comparison of cAMP responses measured with the two sensors, it is paramount that their sensitivity to cAMP is similar. Therefore, BRET dose-response relationship to cAMP was tested in lysates from HEK293 cells transfected with either CAMYEL or PDE2-CAMYEL. There was no significant difference in the activation constants of CAMYEL and PDE2-CAMYEL (P = 0.12; Figure 2).

### Cytosolic and membrane-associated PDEs in HEK293 cells

**Figure 3:**
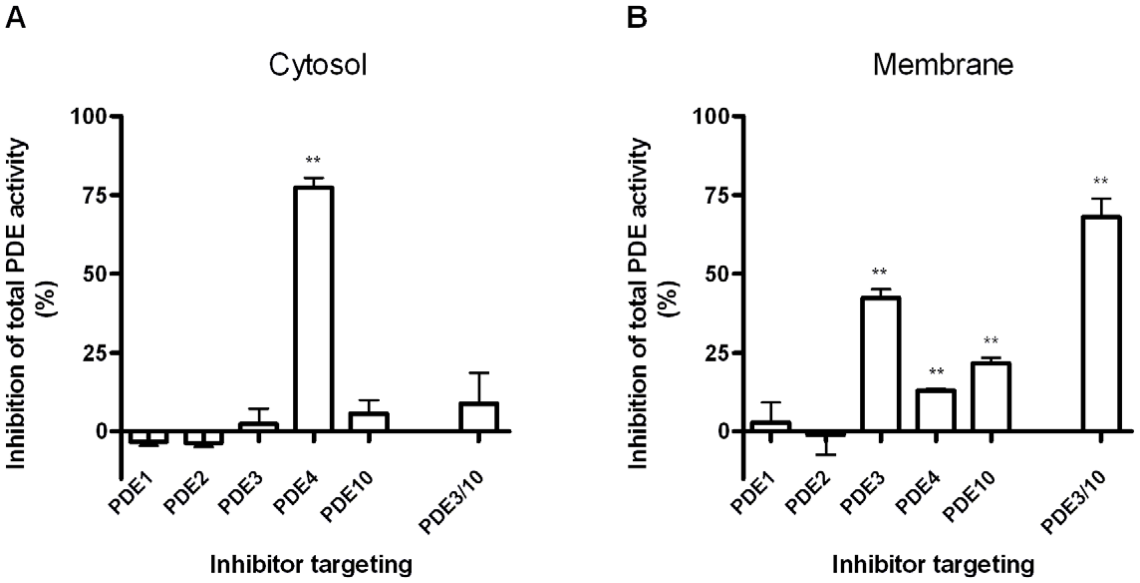
cAMP PDE activity in HEK293 cytosol and membrane fractions. Lysate from either the cytosolic fraction (A) or the membrane fraction (B) was tested in the PDE activity with 1 µM cAMP. Selective PDE inhibitors were added alone or in combination as indicated. The results represent the mean ± SEM of three experiments. Compounds and concentrations used: PDE1i, 250 nM (see M&M section); PDE2i, 20 nM BAY 60-7550; PDE3i, 400 nM cilostamide; PDE4i, 50 nM roflumilast; PDE10i, 300 nM MP-10. The ratio of total cAMP PDE activity in the cytosolic fraction and membrane fraction compared to whole cells was 40.4±5.3% and 59.6±5.3%, respectively.

We estimated the relative contribution of different PDEs in PDE activity assays on lysates from HEK293 cells with selective PDE inhibitors targeting PDE1, PDE2, PDE3, PDE4 and PDE10. We performed the PDE activity assays on separated cytosol and membrane fractions (Figure 3) in order to test whether PDEs localized to either membrane or cytosol have differential effects on cAMP control. Of the PDEs tested, PDE4 was the only significant contributor to cAMP PDE activity in the cytosolic fraction (Figure 3A). In contrast, the membrane fraction was dominated by PDE3 (42%) and to a smaller extent PDE10 (22%), with only a small, but significant (13%), contribution of PDE4 (Figure 3B). In conclusion, we found that PDE4 activity dominated in the cytosolic fraction of HEK293 cells whereas PDEs 3 and 10 dominated in the membrane fraction.

### BRET measurements of cAMP response to forskolin or PGE1

Having determined which PDEs dominate in membrane and cytosolic fractions, we next wanted to determine whether PDEs localized to membrane or cytosol had differential effects on cAMP concentration in these compartments. Furthermore, we tested two different stimulation paradigms to see if they influenced compartmentalization and PDE control of the cAMP response: Forskolin, which directly activates adenylyl cyclases, and prostaglandin E1 (PGE1), which activates adenylyl cyclases indirectly through GPCR stimulation.

**Figure 4:**
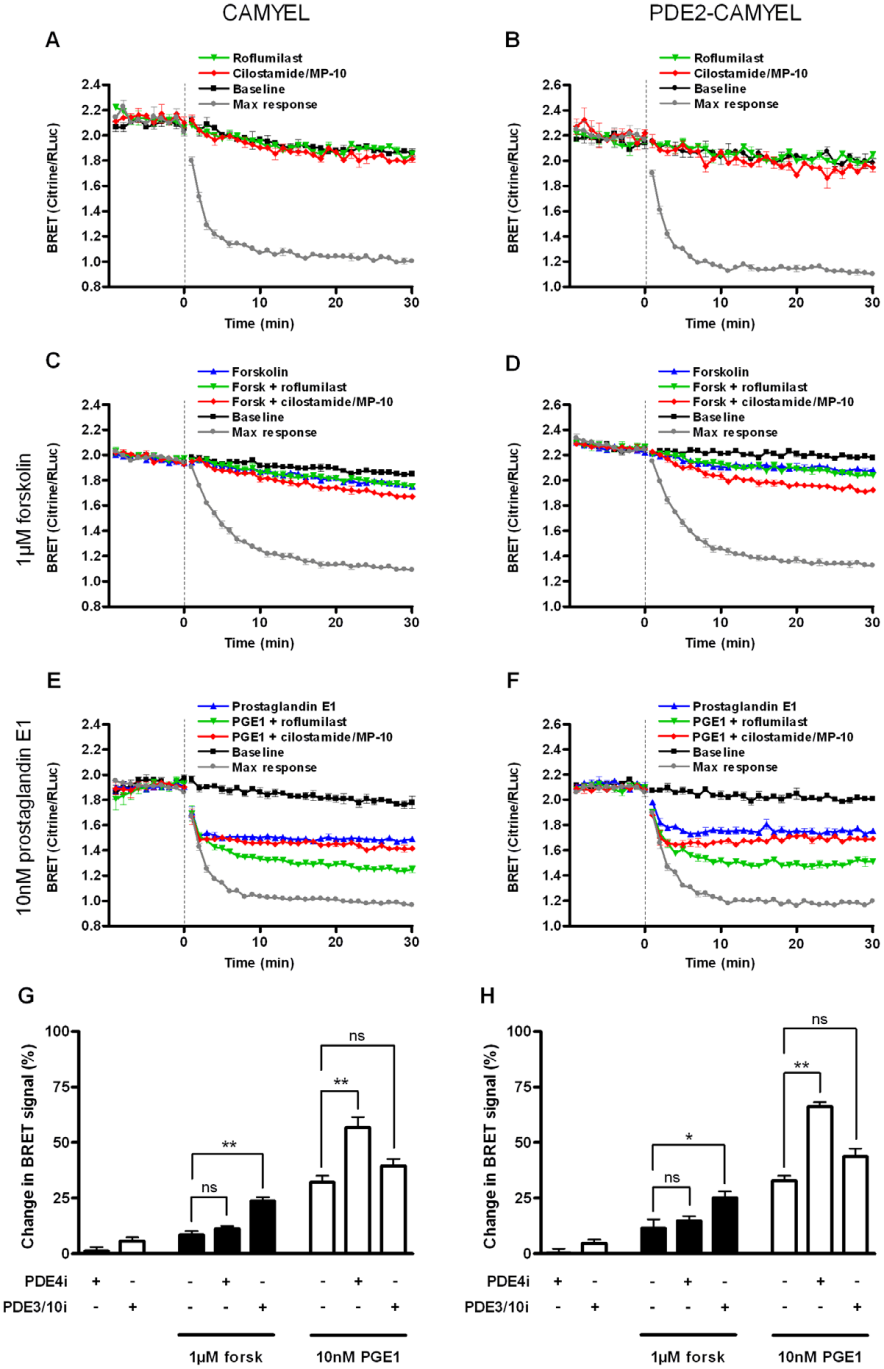
Effect of selective PDE inhibitors on cAMP response in HEK293 cells. Changes in BRET signal were measured in HEK293 cells transfected with either CAMYEL or PDE2-CAMYEL. (A and B) BRET was measured in response to addition of PDE inhibitors. 50 nM roflumilast or 400 nM cilostamide and 300 nM MP-10 was added at time 0. (C–F) BRET was measured in response to stimulation with 1 µM forskolin (C, D) or 10 nM PGE1 (E, F). Forskolin or PGE1 was added at time 0 alone or in combination with 50 nM roflumilast or 400 nM cilostamide and 300 nM MP-10. For baselines, buffer was added, and for maximum response, 10 µM forskolin plus all three PDE inhibitors were added. Each point represents the mean ± SEM of triplicate determinations in a representative experiment. (G and H) Summary of BRET measurements. Relative BRET responses are averaged for the time period 21–30 min after addition of drugs. The results represent the mean ± SEM of three experiments. ns (not significant), P>0.05; *, 0.01<P<0.05; **, 0.001<P<0.01.

HEK293 cells were transfected either with the cytosolic BRET sensor CAMYEL or the membrane-bound PDE2-CAMYEL. Following baseline measurement, cells were stimulated with 1 µM forskolin or 10 nM PGE1, either alone or together with a PDE4 inhibitor or a combination of PDE3 and PDE10 (PDE3/10) inhibitors. Data from representative experiments for the different treatments are shown in Figure 4A–F, where each point indicates the mean ± SEM of triplicate determinations in each experiment. For all treatments except PDE inhibitors alone, BRET signal decreased in response to treatment (indicating increasing cAMP levels), reaching a plateau after 3–10 minutes.

In order to facilitate comparison of BRET responses between experiments, relative responses were calculated by comparison of the average response for the time period 21–30 min after addition of drugs for each treatment to untreated cells and maximum response in the same experiment. Mean ± SEM relative responses from three independent experiments are shown in Figure 4G and H.

Addition of PDE inhibitors alone to the HEK293 cells did not result in significant changes in the BRET signal for either sensor (Figure 4A and B, results summarized in G and H).

The relative response to 1 µM forskolin was 8.4±1.9% for cells transfected with CAMYEL and 11.4±4.1% for those transfected with PDE2-CAMYEL (Figure 4C and D, results summarized in G and H). Addition of PDE3/10 inhibitors increased the response up to three times for both the cytosolic and membrane targeted BRET variants (to 23.5±2.0% and 25.2±2.8%, respectively). In contrast, PDE4 inhibition did not significantly increase the cAMP response to 1 µM forskolin.

The relative response to 10 nM PGE1 was 32.1±2.9% for CAMYEL and 32.8±2.2% for PDE2-CAMYEL (Figure 4E and F, results summarized in G and H). The modulation of the response by PDE inhibitors after stimulation with PGE1 was different from that observed after stimulation with forskolin. Thus, the PDE4 inhibitor augmented the response to 56.8±4.7% and 66.2±2.0% for CAMYEL and PDE2-CAMYEL, respectively, whereas PDE3/10 inhibitors had no significant effect on PGE1-induced cAMP increase measured with either of the BRET sensors.

When the relative responses of the membrane-bound PDE2-CAMYEL sensor and the cytosolic CAMYEL sensor for the six different conditions tested in Figure 4 were compared, we found no significant differences between the response measured with PDE2-CAMYEL and that measured with CAMYEL. However, all 6 comparisons showed a trend towards stronger response with PDE2-CAMYEL, suggesting that membrane-proximal cAMP levels may be slightly higher than in bulk cytosol. We saw no difference in the time to reach half-maximal BRET response for the two sensors for any of the treatments, including the maximal stimulation, suggesting that the rate of cAMP increase is similar near the plasma membrane and in the cytosol (results not shown).

In conclusion, there was a consistent trend towards a stronger BRET response with the membrane-proximal PDE2-CAMYEL sensor than the cytosolic CAMYEL sensor suggesting a slightly higher cAMP increase near the membrane. However, while PDE inhibition increased the cAMP response, there was no evidence of a differential effect of inhibiting membrane-associated and cytosolic PDEs on cAMP measured in either compartment. Surprisingly, inhibition of PDE3/10 augmented the cAMP response to 1 µM forskolin, but not that of 10 nM PGE1, while the opposite was observed for inhibition of PDE4.

### Relative importance of different PDEs depends on the level of stimulation rather than mode of stimulation

**Figure 5:**
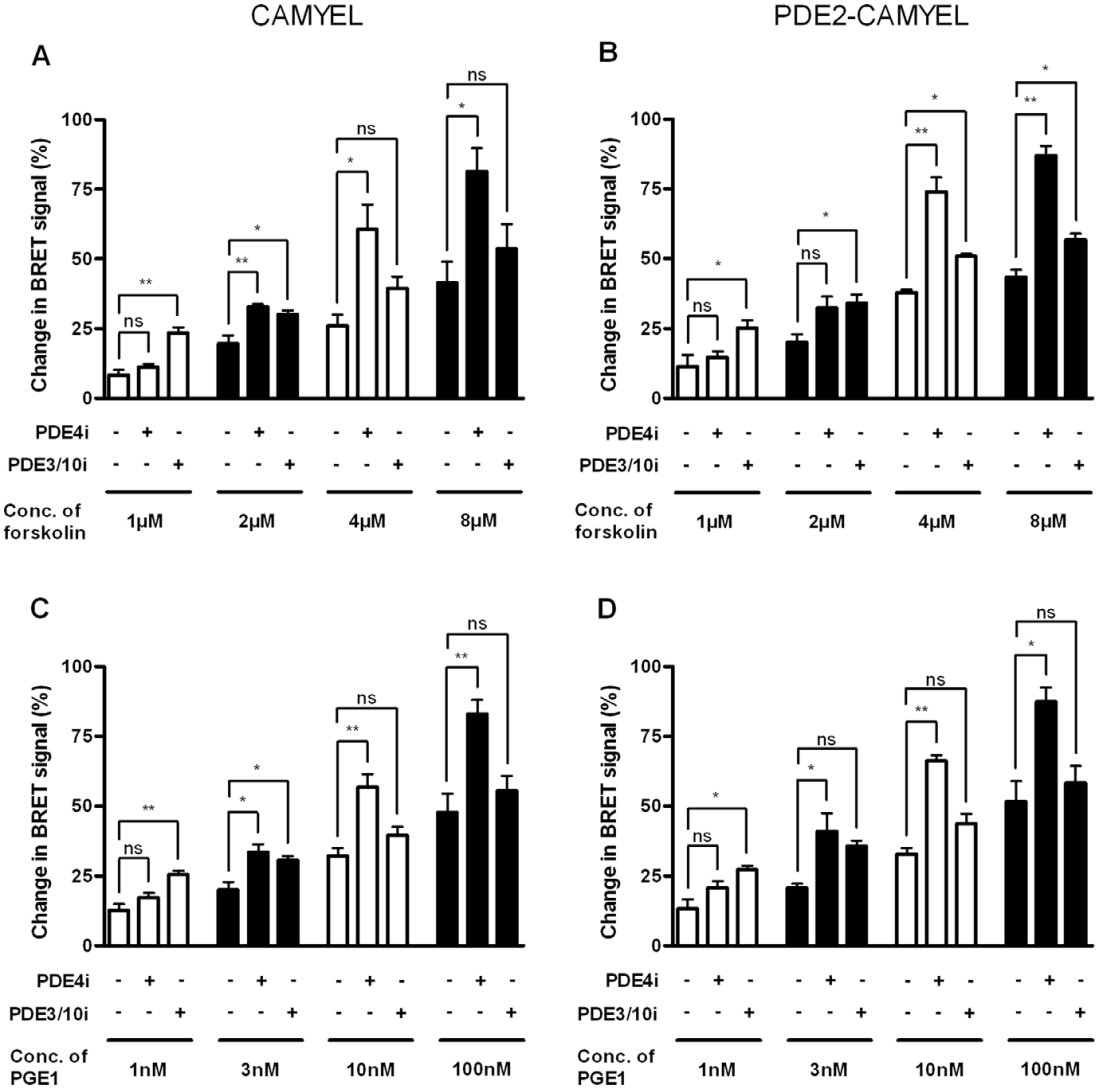
Different PDEs dominate at different cAMP concentrations. Average relative BRET response 21–30 min after addition of forskolin (A, B) or PGE1 (C, D) in combination with PDE inhibitors to HEK293 cells transfected with CAMYEL or PDE2-CAMYEL. Different concentrations of forskolin or PGE1 was added alone or in combination with 50 nM roflumilast or 400 nM cilostamide and 300 nM MP-10 as indicated. From curves similar to those shown in Figure 4, the change in BRET signal was calculated relative to the maximum response (10 µM forskolin plus all three PDE inhibitors). The results represent the mean ± SEM of three experiments. ns (not significant), P>0.05; *, 0.01<P<0.05; **, 0.001<P<0.01.

The differential modulation by PDE4 and PDE3/10 inhibitors of the cAMP response to 1 µM forskolin and 10 nM PGE1 may either reflect a difference in PDE cAMP control of the two modes of stimulation or be a result of the strength of activation - 10 nM PGE1 alone gave a 3–4 fold larger increase in BRET response than 1 µM forskolin. To address this question, we tested a range of forskolin and PGE1 concentrations in combination with PDE inhibitors. As in the previous experiment, cAMP was measured both by the plasma-membrane and in cytosol to evaluate if there was differential regulation of cAMP, dependent on cAMP concentration. Mean ± SEM relative responses from three independent experiments for these conditions are shown in Figure 5, while time-resolved absolute changes in BRET signal are shown in Figure S1 and S2.

As expected, the BRET response increased with increasing concentrations of forskolin and PGE1. The BRET response to 1 nM PGE1 was comparable to that induced by 1 µM forskolin, and, in both cases, the PDE4 inhibitor did not significantly increase the BRET response, while it was increased 2–3 folds by PDE3/10 inhibitors. Increasing concentrations to 3 nM PGE1 and 2 µM forskolin approximately doubled the BRET response and changed the impact of PDE inhibition in both cases so both PDE4 and PDE3/10 inhibitors gave an increase in BRET response. This trend continued so at even higher levels of stimulation, PDE4 inhibitor robustly increased cAMP response, while only small or insignificant effects was observed for PDE3/10 inhibitors.

There was a larger response with the membrane-proximal PDE2-CAMYEL sensor than the cytosolic CAMYEL in 23 out of 24 tested conditions though the difference was only significant for one of them (4 µM forskolin without PDE inhibitors). Again, no difference in the time to reach half-maximal BRET response for the two sensors was observed (results not shown).

In summary, independent of the method of stimulation, PDE3/10 inhibition predominantly increases cAMP response to adenylyl cyclase stimulation at low levels of stimulation, while PDE4 inhibition predominantly increases cAMP response at higher levels of stimulation. The BRET responses with the membrane-proximal PDE2-CAMYEL sensor are generally slightly stronger than those of the cytosolic CAMYEL sensor, suggesting a slightly higher cAMP response near the membrane. There was no evidence for differential effect of membrane associated PDEs compared to cytosolic PDEs in the regulation of cAMP in the same areas.

## Discussion

In this study, we have measured intracellular cAMP concentrations in living HEK293 cells with the BRET sensor CAMYEL [17] and a novel membrane-targeted variant that we have made by fusing CAMYEL to the membrane-targeting motif from PDE2. Responses from differently targeted molecular sensors can only be directly compared when they are unimolecular and have very similar or identical cAMP affinity as we have shown for our BRET sensors. Two previous studies have used such validated sensors in HEK293 cells. Terrin *et al.* [13] observed a maximal cAMP response to 1 µM PGE1 of 31.4±1.2% with a membrane-targeted FRET sensor compared to 23.5±1.4% with a cytosolic sensor, demonstrating a significantly higher cAMP concentration at the plasma membrane. DiPilato *et al.* [12] observed a maximal cAMP response to 10 µM isoproterenol of 18.3±1.2% with a membrane-targeted FRET sensor compared to 16.8±1.0% with a cytosolic sensor, a trend to higher cAMP at the plasma membrane. In our study, most individual comparisons between the cAMP BRET response at the plasma membrane and in the cytosol were statistically insignificant. However, the cAMP BRET response at the plasma membrane was higher than in the cytosol in 23 out of 24 comparisons, which is highly significant. So in accordance with previous studies using FRET sensors [12], [13], our study with BRET sensors suggests a small gradient of cAMP from the plasma membrane in stimulated HEK293 cells.

The best supported mechanism for achieving cAMP compartmentalization is that the PDEs are compartmentalized as well; therefore, a detailed analysis of PDE activity in the model system is critical. In an earlier study of cAMP PDE activity in HEK293B2 cells, PDEs 3, 4B and 4D were found to be major PDEs, but only PDE3 and PDE4 inhibitors were used in the analysis [20]. The PDE activity analysis in our study provides additional details by testing PDE activity in subcellularly fractionated lysates rather than total lysate and by testing additional selective PDE inhibitors. We found that PDE10A also contributes to PDE activity in HEK293 cells and that PDEs 3 and 10 dominate PDE activity in the membrane fraction while PDE4 is the dominant PDE in the cytosolic fraction.

The clear subcellular separation of the PDEs permitted us to compare the impact of inhibition of the membrane associated PDEs 3 and 10 to the predominantly cytosolic PDE4 in living cells. We did not observe any differential effects on cAMP measured with the cytosolic and the membrane-bound BRET sensor with either group of inhibitors. The apparent lack of differential regulation of cAMP by the PDEs was observed both for forskolin and PGE1 cAMP induction. The sensitivity of the analysis is of course likely to be limited by the small subcellular differences in cAMP concentrations observed in the small HEK293 cells. Furthermore, it should be noted that PDE4 is also present at the membrane and that the relative contribution of PDEs to cAMP control may be different in living cells and lysate due to for example different localization of the PDEs within the membrane compartment. Our data are in apparent contrast to data obtained by Terrin *et al.* [13]: In HEK293 cells stimulated with 1 µM PGE1, siRNA-mediated knockdown of cytosolic PDE4D was found to invert the cAMP gradient from membrane to cytosol, whereas knockdown of the membrane-bound PDE4B had no effect [13]. The apparent discrepancy with our result may be due to the difference between the acute inhibition with inhibitors and siRNA-mediated knockdown, which is long-lasting and may lead to compensatory changes. Supporting a difference between genetic knockdown and chemical inhibition, they observed no effect on the cAMP gradient in response to treatment with the PDE4 inhibitor rolipram, while it was abolished when both active PDE4 isoforms PDE4B and PDE4D were knocked down simultaneously with siRNA [13].

Irrespective of the localization of the cAMP BRET sensor, we found that PDE3/10 inhibitors more than doubled the BRET signal in HEK293 cells at low levels of adenylyl cyclase stimulation, but had low or non-significant effects at higher levels of adenylyl cyclase stimulation. In contrast, the PDE4 inhibitor had no significant effect on intracellular cAMP concentrations at low levels of adenylyl cyclase stimulation, while it strongly enhanced the BRET signal at higher levels of adenylyl cyclase stimulation. The different subcellular localizations of the PDEs may contribute, but the different K_M_ of the PDEs is a more likely explanation. Reported estimates of cAMP K_M_ for PDE3 and PDE10 are 0.15–0.38 µM [21]–[23] and 0.05–0.2 µM [24], [25], respectively, whereas it is 1.2–5.2 µM for PDE4, depending on the subtype [26]. The higher K_M_ of PDE4 means that the relative activity of PDE4 will increase at higher cAMP concentrations, since the PDE3 and PDE10 enzymes will saturate faster. The effects of PDE3/10 and PDE4 inhibitors on intracellular cAMP concentrations as measured with the BRET sensors are approximately equal at concentrations of 2 µM forskolin or 3 nM PGE1. At these concentrations, the effect of the stimulants alone results in a BRET change of around 20%, which from the *in vitro* characterization of the BRET sensors (Figure 2) can be calculated to correspond to a cAMP concentration of just over 1 µM. At this cAMP concentration, total PDE4 activity was found to be similar to total activity of PDE3 and PDE10.

Although there are some recent reports of other systems where substrate affinity seems to be critical for determining PDE control of cAMP [27], [28], the influence of the different affinities of PDEs for cAMP on the control of cAMP concentration in living cells has not received much attention. The data presented here suggest that in HEK293 cells, the enzymatic properties of the PDEs are more important than their subcellular localization in determining their effect on cAMP concentration at the plasma membrane and in the cytosol.

## Materials and Methods

### Reagents

Prostaglandin E_1_, forskolin, and cilostamide were purchased from Sigma, BAY 60-7550 was purchased from Alexis, and Coelenterazine-h from Interchim. MP-10 and roflumilast were synthesized by H. Lundbeck A/S. The PDE1 inhibitor used in this study was 5-(4-Diethylamino-benzyl)-1-methyl-3-propyl-1,6-dihydro-pyrazolo[4,3-d]pyrimidin-7-one [29], which was also synthesized by H. Lundbeck A/S.

### Cell culture and transfection

HEK293 cells were obtained from American Type Culture Collection and cultured in Dulbecco's modified Eagle medium with GlutaMAX (Invitrogen) supplemented with 10% fetal bovine serum, 100 U/ml penicillin, and 100 µg/ml streptomycin. Transfection was done with FuGENE 6 (Roche) according to the manufacturer's instructions. Lysis, imaging and BRET measurements were performed after 24–48 hours.

### PDE activity assays

HEK293 cells were lysed in 50 mM Tris buffer, pH 8.0, with 1 mM MgCl_2_ and 1% Complete protease inhibitor cocktail (Roche) and separated into cytosol and membrane fraction. After centrifugation for 30 min at 20,000× *g*, the supernatant was removed and 0.1% Triton X100 added (cytosolic fraction). The pellet was resuspended in the same buffer, but with 0.5% Triton X100 added, the centrifugation was repeated, and the supernatant removed (membrane fraction). PDE activity was measured using a scintillation proximity assay (SPA)-based method as previously described [30]. Reported IC_50_s for PDE inhibitors used (primary target): 5-(4-Diethylamino-benzyl)-1-methyl-3-propyl-1,6-dihydro-pyrazolo[4,3-d]pyrimidin-7-one (PDE1), 38 nM [29]; BAY 60-7550 (PDE2), 4.7 nM [31]; cilostamide (PDE3), 27–50 nM [32]; roflumilast (PDE4), 0.8 nM [33]; MP-10 (PDE10), 0.18 nM [34].

### BRET sensor constructs

The BRET sensor CAMYEL (pcDNA3L-His-CAMYEL) was purchased from ATCC. For generation of the membrane-targeted variant PDE2-CAMYEL, DNA encoding the N-terminal membrane targeting domain of PDE2A3 (amino acids 1–196) was PCR amplified using the primer pair 5′-GTG CAA GCT TAT GGG GCA GGC ATG CGG CCA-3′ and 5′-CGT GTA CAG CTG CTG CAG GAC CTG CAC C-3′ followed by insertion into pcDNA3L-His-CAMYEL using HindIII and BsrGI.

### Subcellular localization of BRET sensor

Western blotting: HEK293 cells transfected with BRET sensor constructs were harvested, lysed and fractionated into cytosolic and membrane fractions as described for the PDE activity assays. The fractions were subjected to denaturing SDS/PAGE on 4–12% Bis-Tris gel (Invitrogen) and subsequent Western blotting on polyvinylidene difluoride membranes (Millipore). Rabbit polyclonal anti-GFP antibody (Abcam) was used as primary antibody. Swine anti-rabbit conjugated with horseradish peroxidase (DAKO) was used as secondary antibody. The blot was developed using the SuperSignal West Dura kit (Pierce).

Imaging: HEK293 cells that had been transfected with either BRET sensor construct on glass coverslips were fixated with 4% paraformaldehyde. Confocal images were acquired on an Eclipse TE300 microscope (Nikon) equipped with the Bio-Rad Radiance2000 confocal system. Cells were excited using the 488 nm line of the Krypton/Argon laser for imaging Citrine.

### Activation constants of BRET sensors

HEK293 cells expressing CAMYEL or PDE2-CAMYEL were harvested and lysed in PBS∶H_2_O, 1∶2 with 0.5% Triton X100 and 1% Complete protease inhibitor cocktail (Roche). After centrifugation at 20,000× *g* for 30 min, the supernatant was removed and the ionic concentrations adjusted towards intracellular levels (40 mM Hepes, pH 7.2, 140 mM KCl, 10 mM NaCl, 1.5 mM MgCl_2_). Coelenterazine-h was added to a final concentration of 5 µM together with different concentrations of cAMP. Emission from RLuc and Citrine was measured simultaneously at 460 nm and 535 nm in an EnVision Multilabel Reader (PerkinElmer). Apparent activation constants were determined by fitting the obtained data to a sigmoidal dose-response curve (GraphPad Prism 4).

### BRET assays on living cells

HEK293 cells were seeded at 1×10^6^ cells/well in 6-well plates. After 24 h, cells were transfected with CAMYEL or PDE2-CAMYEL. 24 h after transfection, cells in each well were washed in Hank's balanced salt solution (HBSS), detached using three drops of trypsin (0.05% Trypsin/EDTA, Invitrogen), suspended in 1.5 ml HBSS and 80 µL/well were transferred to white 96-well half area plates (Corning). After 1 h of serum starvation in incubator, 10 µL coelenterazine-h in HBSS was added to a final concentration of 5 µM and emission from RLuc and Citrine was measured simultaneously at 460 nm and 535 nm every minute in an EnVision Multilabel Reader (PerkinElmer). BRET signal was calculated as the ratio between emission at 535 nm and emission at 460 nm. Stimulation was initialized by addition of 10 µl forskolin/PGE1 and PDE inhibitor(s) diluted in HBSS to 10× final concentration. All experiments included untreated cells and cells treated with 10 µM forskolin together with PDE3, 4 and 10 inhibitors that was used to estimate and compare maximum response.

## Supporting Information

Figure S1 **Changes in cAMP concentration in response to forskolin.** BRET was measured in HEK293 cells transfected with either CAMYEL or PDE2-CAMYEL. 1 µM (A, B), 2 µM (C, D), 4 µM (E, F) or 8 µM (G, H) forskolin was added at time 0 alone or in combination with 50 nM roflumilast or 400 nM cilostamide and 300 nM MP-10. For baselines, buffer was added, and for maximum response, 10 µM forskolin plus all three PDE inhibitors were added. Each point represents the mean ± SEM of triplicate determinations. Data represents one of three experiments summarized in Figure 5A and B.
(TIF)

Figure S2 **Changes in cAMP concentration in response to prostaglandin E1.** BRET was measured for HEK293 cells transfected with either CAMYEL or PDE2-CAMYEL. 1 nM (A, B), 3 nM (C, D), 10 nM (E, F) or 100 nM (G, H) PGE1 was added at time 0 alone or in combination with 50 nM roflumilast or 400 nM cilostamide and 300 nM MP-10. For baselines, buffer was added, and for maximum response, 10 µM forskolin plus all three PDE inhibitors were added. Each point represents the mean ± SEM of triplicate determinations. Data represents one of three experiments summarized in Figure 5C and D.
(TIF)
